# Enhancing end-stage renal disease outcome prediction: a multisourced data-driven approach

**DOI:** 10.1093/jamia/ocaf118

**Published:** 2025-08-06

**Authors:** Yubo Li, Rema Padman

**Affiliations:** Heinz College of Information Systems and Public Policy, Carnegie Mellon University, Pittsburgh, PA 15213, United States; Heinz College of Information Systems and Public Policy, Carnegie Mellon University, Pittsburgh, PA 15213, United States

**Keywords:** chronic kidney disease, end-stage renal disease, machine learning, clinical and claims data integration, predictive modeling

## Abstract

**Objectives:**

To improve prediction of chronic kidney disease (CKD) progression to end-stage renal disease (ESRD) using machine learning (ML) and deep learning (DL) models applied to integrated clinical and claims data with varying observation windows, supported by explainable artificial intelligence (AI) to enhance interpretability and reduce bias.

**Materials and Methods:**

We utilized data from 10 326 CKD patients, combining clinical and claims information from 2009 to 2018. After preprocessing, cohort identification, and feature engineering, we evaluated multiple statistical, ML and DL models using 5 distinct observation windows. Feature importance and SHapley Additive exPlanations (SHAP) analysis were employed to understand key predictors. Models were tested for robustness, clinical relevance, misclassification patterns, and bias.

**Results:**

Integrated data models outperformed single data source models, with long short-term memory achieving the highest area under the receiver operating characteristic curve (AUROC) (0.93) and F1 score (0.65). A 24-month observation window optimally balanced early detection and prediction accuracy. The 2021 estimated glomerular filtration rate (eGFR) equation improved prediction accuracy and reduced racial bias, particularly for African American patients.

**Discussion:**

Improved prediction accuracy, interpretability, and bias mitigation strategies have the potential to enhance CKD management, support targeted interventions, and reduce health-care disparities.

**Conclusion:**

This study presents a robust framework for predicting ESRD outcomes, improving clinical decision-making through integrated multisourced data and advanced analytics. Future research will expand data integration and extend this framework to other chronic diseases.

## Introduction

Chronic kidney disease (CKD) is a complex, multimorbid condition marked by a gradual decline in kidney function, which can ultimately progress to end-stage renal disease (ESRD).^1^ With a global prevalence of CKD ranging from 8% to 16%, and estimates suggesting that around 5%-10% of individuals diagnosed with CKD eventually reach ESRD,^2^ they represent a major public health challenge, particularly due to its strong associations with diabetes and hypertension.^3^ Chronic kidney disease progression is classified into 5 stages, culminating in ESRD, where kidney function drops to 10%-15% of normal capacity, necessitating dialysis or transplantation for patient survival.^1^ The economic impact of CKD is significant, with a relatively small proportion of Medicare CKD patients in the United States contributing to a disproportionately high share of Medicare expenses, particularly when they progress to ESRD. Additionally, more than one-third of ESRD patients are readmitted within 30 days of discharge, underscoring the critical need for early detection and management of CKD to prevent its progression to ESRD and to reduce health-care costs.^4^

Previous CKD progression prediction efforts used either electronic health record (EHR) clinical data^5^ or administrative claims.^6^^,^^7^ These approaches often use limited features, not fully capturing CKD progression complexity. Sharma et al^8^ developed a claims-based model to identify CKD patients at hyperkalemia risk, while Krishnamurthy et al^6^ predicted CKD onset using comorbidities and medications. Claims data typically lack clinical data’s granularity. Tangri et al^9^ used age, gender, and estimated glomerular filtration rate (eGFR)^10^ to predict ESRD progression, and Sun et al^11^ utilized serum creatinine and urine protein levels for high-risk patient identification. Models using only clinical data may miss complete health-care system interactions, struggle with missing data and inconsistent recording, and overlook socioeconomic factors and health-care utilization patterns, limiting their applicability across diverse populations.

This study bridges a critical gap by developing a framework that utilizes integrated clinical and claims data rather than isolated data sources. By minimizing the observation window needed for accurate predictions, our approach balances clinical relevance with patient-centered practicality. This integration enhances both predictive accuracy and clinical utility, enabling more informed decision-making to improve patient outcomes.

## Objective

**Figure 1.**
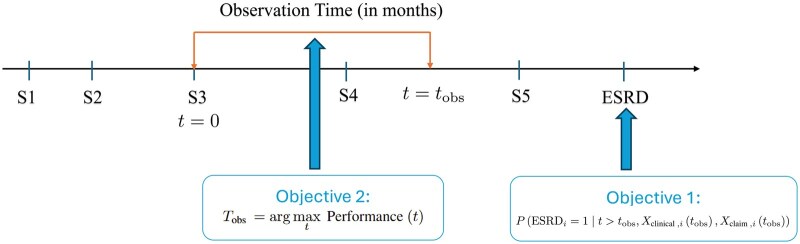
A timeline illustration of observation windows for CKD progression to ESRD. The observation starts at CKD stage 3. Objective 1 estimates the probability of ESRD occurrence after the observation window, using clinical and claims data within the observation window, while objective 2 identifies the optimal observation window that maximizes predictive performance. Abbreviations: CKD, chronic kidney disease; ESRD, end-stage renal disease.

This research evaluates predictive models for CKD progression to ESRD using integrated clinical and claims data. Figure 1 illustrates our study design, where the observation window begins at the initial diagnosis of CKD stage 3 (*t* = 0). Although patients may progress through subsequent CKD stages (stages 4 and 5) during this observation period, our cohort specifically excludes those developing ESRD within this timeframe. This ensures that predictive modeling utilizes only pre-ESRD data for forecasting future ESRD onset.

- Our primary objective, ESRD risk prediction, aims to estimate the probability that a patient diagnosed with CKD stage 3 will develop ESRD after the observation window, represented as follows:
  $$P({\mathrm{ESRD}}_{i}=1|t>t_{\mathrm{obs}},X_{\mathrm{clinical},i}(t_{\mathrm{obs}}),X_{\mathrm{claim},i}(t_{\mathrm{obs}})),$$

where $X_{\mathrm{clinical},i}\;(t_{\mathrm{obs}})$, $X_{\mathrm{claim},i}\;(t_{\mathrm{obs}})$ denote the clinical and claims data observed up to time $t_{\mathrm{obs}}$ for patient i.

- To identify the optimal observation window *T*_obs_ that provides the best performance across all candidate windows $t_{\mathrm{obs}}$ (6, 12, 18, 24, and 30 months), ensuring accurate predictions while minimizing the length of the observation window required:
  $$T_{\mathrm{obs}}=\arg\underset{t\in \{6,12,18,24,30\}}{\max}\mathrm{Performance}(t),$$

where Performance(t) is evaluated based on key predictive metrics: F1 score,^12^ area under the receiver operating characteristic curve (AUROC),^13^ and area under the precision-recall curve (AUPRC).^14^

## Data sources

This study utilized 2 integrated datasets: administrative claims data and clinical data. The claims dataset includes patient health-care interactions, spanning from 2009 to 2018, containing diagnosis codes, treatment records, and health-care costs for individuals diagnosed with CKD. The clinical dataset from EHRs contains laboratory results, patient demographics, diagnostic details, and medication records, which was truncated to match the 10-year span of the claims data for consistency of integration. See Appendix S1 for data details.

## Methods

**Figure 2.**
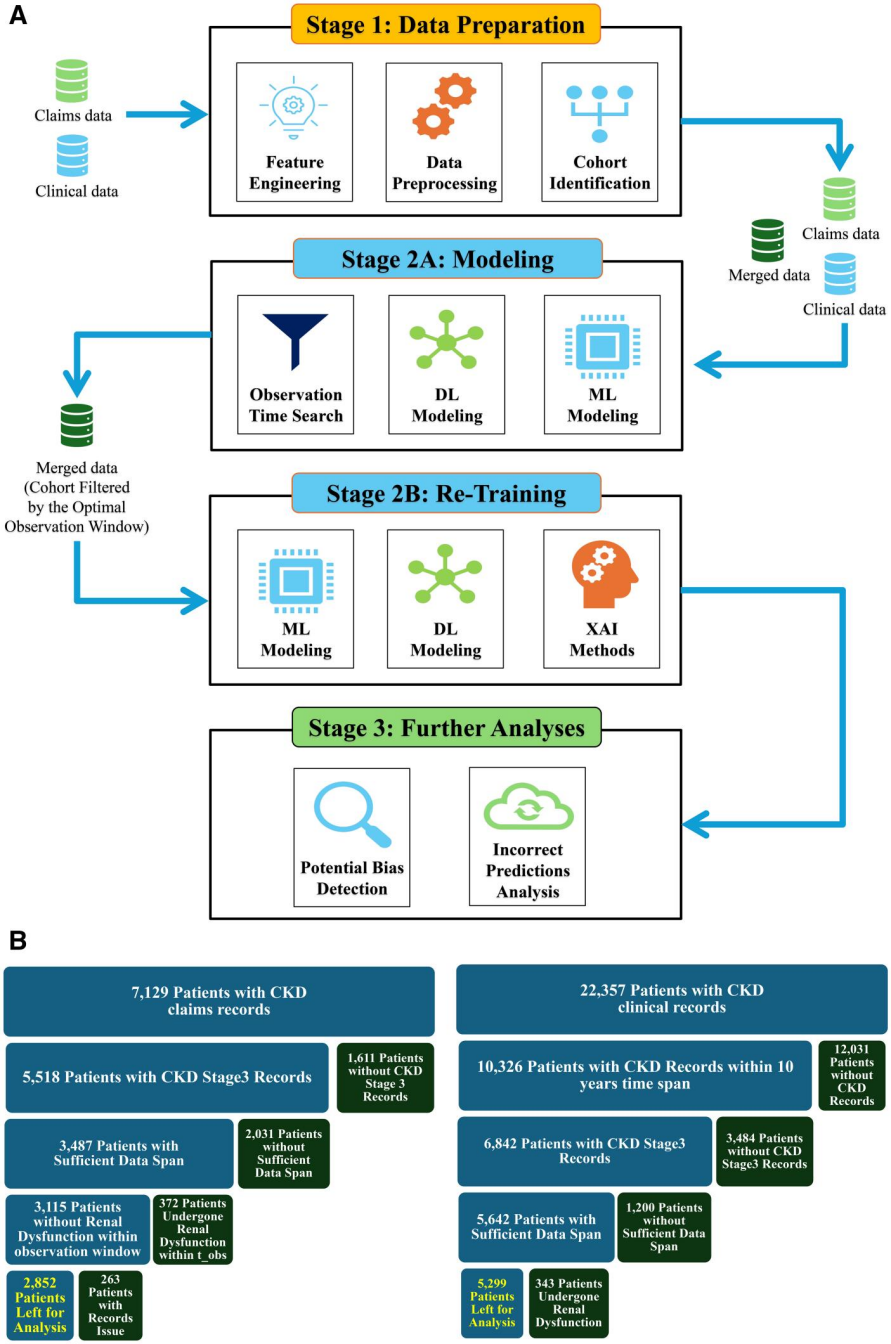
Framework for CKD progression prediction. (A) Three-stage pipeline for predicting CKD progression to ESRD. Stage 1: feature engineering, data preprocessing, and cohort identification using clinical and claims data. Stage 2: (2A) systematic evaluation of observation windows with ML/DL models; (2B) retraining the optimal model on the filtered cohort from the optimal observation window, followed by explainable artificial intelligence (AI) analysis. Stage 3: Additional analyses including bias detection and misclassification assessment. (B) Multistep cohort curation process for longitudinal CKD analysis. Starting with initial selection of patients at CKD stage 3, an adjustable observation window (18 months shown) was applied. Only patients with complete clinical and claims data throughout this period were retained, while those progressing to ESRD within the observation window were excluded, ensuring a cohort for analysis (highlighted in yellow) with uninterrupted longitudinal records. Abbreviations: CKD, chronic kidney disease; DL, deep learning; ESRD, end-stage renal disease; ML, machine learning.

As illustrated in Figure 2A, our comprehensive methodology for predicting CKD progression to ESRD consists of 3 primary stages: data preparation, modeling, and additional analyses. Within the modeling stage, we employ a 2-phase analytical framework that first systematically evaluates various predictive models across multiple observation windows (but the same patient cohort for consistent comparisons) to determine optimal performance, and subsequently retrains the most effective model on the cohort for each observation window for practical clinical application. This framework bridges statistical rigor and clinical relevance, facilitating actionable insights for nephrologists managing patients with CKD stage 3 and above.

### Data preparation

#### Data preprocessing

We removed duplicate records to prevent redundancy and bias in the analysis. Entries lacking CKD diagnoses were excluded to maintain relevance to study objectives, along with claims containing negative values (likely data entry errors). The cleaned claims dataset comprised 5 317 178 claims across 7129 unique patients, while the refined clinical dataset included 433 421 laboratory records for 10 326 patients. These datasets were integrated using unique patient IDs to provide a comprehensive view of each patient’s medical and claims history.

To address missing data, we employed multiple imputation via chained equations.^15^ Continuous numerical features were standardized, and skewed variables were log-transformed^16^ to stabilize variance and mitigate outliers. For laboratory values missing unit specifications, we used distribution detection and adjustment^17^ to identify outliers and harmonize units across records (medical expert-advised ranges in Appendix S2).

#### Cohort identification

As depicted in Figure 2B, cohorts were curated through a multistep process: patients at CKD stage 3 were selected and an observation window—18 months in the example, but adjustable per study—was applied. Only individuals with complete clinical and claims data throughout this window were retained, and those who progressed to ESRD within it were excluded, yielding a cohort for analysis.

#### Feature engineering

Feature engineering involved identifying predictive variables essential for modeling. For claims data, we derived 2 main groups of features: cost-based features, including claim counts, aggregate patient costs, cost ranges, and cost standard deviations from inpatient, outpatient, professional, pharmacy, and vision claims; and comorbidity-based features, such as CKD stage 3 duration, emergency department visit frequency, and critical comorbidities (eg, hypertension, diabetes, phosphatemia).

Clinical data features were categorized based on nephrology expert consultations, relevant literature, and dataset characteristics into demographic features, including age, gender, ethnicity, and body mass index (BMI) as established CKD progression indicators; laboratory features comprising essential clinical parameters like eGFR, hemoglobin, phosphorus, serum calcium, and bicarbonate (excluding urine albumin-to-creatinine ratio [UACR]^18^ and sodium due to insufficient data availability); and additional comorbidity features, capturing conditions such as cardiovascular disease, anemia, and metabolic acidosis, which are known to influence CKD outcomes.

Statistical analyses compared variables between patients progressing to ESRD and those who did not. For continuous variables, data were assessed for normality; normally distributed variables were analyzed using independent *t*-tests to compare group means. Nonnormally distributed variables underwent logarithmic transformation during preprocessing before applying *t*-test. Categorical variables were expressed as frequencies (percentages) and compared using chi-squared tests. Statistical significance was set at *P *< .05 for all analyses.

### Modeling and validation

We trained both machine learning (ML) and deep learning (DL) models on stratified train/validation/test splits to preserve class proportions, and applied SMOTE^19^^,^^20^ to the training data to oversample the minority class.

#### ML methods

We employed logistic regression (LR)^21^ as our baseline model for ESRD progression probability estimation. We extended our analysis to random forest (RF)^22^ and XGBoost,^23^ evaluating performance via *k*-fold cross-validation.^24^ Random forest utilizes multiple decision trees to enhance accuracy while reducing overfitting. XGBoost sequentially builds models to correct previous errors, proving highly effective for structured data.

#### DL methods

To develop a robust predictive model for CKD progression to ESRD, we adopted methodologies from prior studies^7^^,^^25^ by constructing longitudinal data representation with 3-month intervals following initial CKD stage 3 diagnosis. These intervals were sequentially indexed as timestamps (0 for months 0-3, 1 for months 3-6, etc.).

This temporal segmentation approach serves several purposes, capturing CKD’s temporal evolution, reflecting its gradual progression, aligning with clinical guidelines recommending periodic nephrologist visits, and accommodating potential time lags between clinical events and their corresponding claims data. These lags, particularly notable for critical events such as transplantation or dialysis initiation, exhibit substantial variability both across and within patient records, complicating consistent correction strategies. Employing 3-month intervals mitigates the impact of these temporal discrepancies, improving model accuracy. Numerical features were aggregated, categorical features encoded binarily to reflect conditions or events, and baseline historical records were analyzed to adjust for preexisting conditions, thereby ensuring accurate representation of CKD progression. This timestamp-based structure allows models to capture disease progression dynamics and identify critical temporal patterns signaling heightened ESRD risk.

We evaluated multiple DL architectures, including convolutional neural networks (CNNs)^26^ for feature extraction; recurrent neural networks (RNNs),^27^ long short-term memory (LSTM) networks,^28^ and gated recurrent units (GRUs)^29^ for modeling temporal dependencies; and temporal convolutional networks (TCNs)^30^ for sequence modeling. These architectures were selected to optimize prediction accuracy of ESRD progression and improve identification of associated risk factors. Detailed implementation information is available in Appendix S4.

To maximize clinical relevance, our analytical framework employs a 2-phase approach (as illustrated in Figure 2A, stage 2). First, we systematically compare all modeling techniques mentioned above across multiple observation windows to identify optimal predictive performance. Second, recognizing that clinicians cannot predetermine a patient’s progression timeline to ESRD in real-world settings, we retrain the best-performing model using the complete cohort with the optimal observation window. This clinically oriented model then undergoes comprehensive explainable AI analysis to identify key predictive features and temporal patterns. This approach bridges the gap between statistical performance and practical clinical implementation, providing actionable insights for nephrologists.

### Explainable AI methods

After identifying the best-performing model and optimal observation window, we employed 2 complementary explainable AI techniques to understand the features driving these predictions. At the cohort level, we utilized feature importance analysis to identify key predictive variables across the population. At the individual level, we applied SHapley Additive exPlanations (SHAP) analysis to provide detailed, patient-specific insights into model predictions.

### Additional analyses

Beyond the core dual-phase predictive framework outlined above, we conducted several additional analyses to enhance the robustness and fairness of our ESRD prediction models. Specifically, we investigated cases of model misclassification to identify underlying patterns and contributing factors, providing deeper insights into prediction limitations. Furthermore, we assessed the influence of recently updated eGFR equation on racial disparities within CKD progression predictions, aiming to ensure clinical fairness and improve the accuracy of our predictive models.

#### Analysis of model misclassifications

We examined predictive probability distributions across our sample set to analyze misclassifications from our optimal model. We evaluated both false positives (type I errors) and false negatives (type II errors) in ESRD progression predictions to identify patterns contributing to incorrect predictions. This error analysis provides insights into model limitations and highlights potential areas for refinement, ultimately improving clinical reliability and decision support.

#### Impact of updated eGFR equations on racial bias in CKD predictions

The 2021 workgroup led by the National Kidney Foundation and American Society of Nephrology recommended an updated CKD-EPI equation that removed race coefficients, addressing concerns about racial bias in eGFR calculations,^31^^,^^32^ such as race-based components lacking biological basis and risking the perpetuation of health-care disparities.^33^^,^^34^

For our study using data from 2009 to 2018, we primarily used the 2009 CKD-EPI equation since this was the standard clinical calculation during the study period and informed actual physician decisions and CKD staging. We additionally applied the 2021 race-free equation in our further analysis section to compare outcomes and assess improvements in prediction accuracy and equity, particularly for minority populations. Note that adopting the updated equation may affect CKD stage classification, potentially altering cohort composition and model predictions.

## Results

### Cohort for analysis and data characteristics

**Figure 3.**
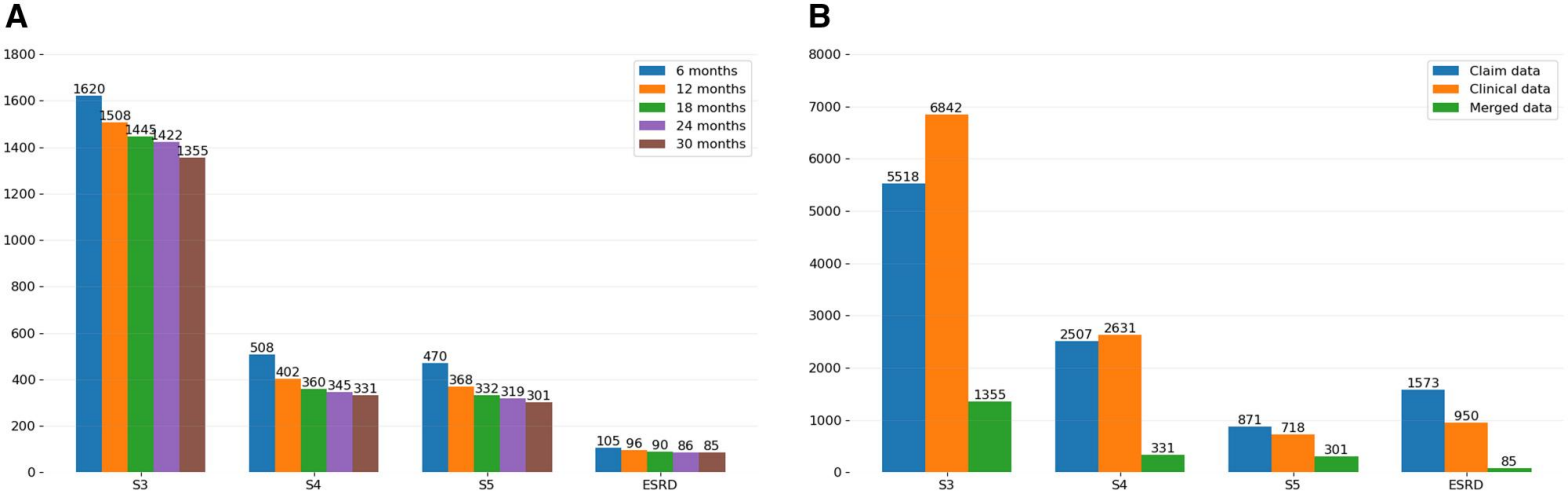
Overview of CKD stage distribution in merged data across observation windows. (A) Variation in CKD stage distribution of merged data as a function of different observation window lengths. (B) Comparison of CKD stage distribution across claims, clinical, and merged data with a 30-month observation window. Abbreviation: CKD, chronic kidney disease.

In this subsection, we present data integration trends and cohort characteristics. Figure 3A shows patient distribution across CKD stages with varying observation windows. Patient numbers decline as windows extend from 6 to 30 months due to insufficient data or ESRD progression within the window. The ESRD columns represent patients who progressed to ESRD after their respective observation windows. While some patient losses may be attributed to mortality, we lack confirmatory data.

To ensure model consistency and fair comparability, we trained and tested our models using the same patient cohort. Recognizing the issue of cohort sizes decreasing as the observation window lengthens, we selected patients who had at least 30 months of follow-up data after their CKD stage 3 diagnosis. We specifically chose 30 months because it represents the smallest cohort across the range of observation windows; thus, patients in this 30-month cohort are also guaranteed to be present in all shorter observation windows. For the training datasets, we utilized this same group of patients but varied the length of the data included according to different observation windows. This approach allowed us to systematically assess how different observation windows affected model performance and to identify the optimal window based on test dataset results.

After requiring ≥30 months of post-stage 3 data, we compared claim-only (*n* = 5518) and clinical-only (*n* = 6842) cohorts to the merged sample. Combining data reduced the cohort to 1422 patients for the 24-month window (optimal) and 1355 for the 30-month window (Figure 3B), reflecting exclusion of those lacking 1 data source and yielding a more comprehensive dataset.

Table 1 summarizes characteristics of our 1422-patient cohort; 86 (6%) progressed to ESRD, while 1336 did not. Mean progression time from CKD stage 3 to ESRD was 4.82 ± 1.82 years. End-stage renal disease patients were younger (69.13 vs 72.04 years, *P* < .001) with significant racial disparities: African Americans showed higher progression rates (14.0% vs 4.5%), while White patients had lower rates (81.4% vs 93.0%, *P* < .001).

**Table 1.** Data characteristics of the patient cohort for analysis (merged data, 24-month observation window, *n* = 1422).

Characteristics	Missing data (%)	Progressed to ESRD (*n* = 86)	Non-progressed to ESRD (*n* = 1336)	*P*
**Demographic**				
Age (years)	0	69.13 ± 12.37	72.04 ± 11.25	*<*.001
Female	0	40 (46.5%)	721 (54.0%)	.2149
Race	0			*<*.001
White		70 (81.4%)	1242 (93.0%)	
African American		12 (14.0%)	60 (4.5%)	
Others		4 (4.6%)	34 (2.5%)	
BMI	4	28.40 ± 5.32	26.40 ± 6.20	*<*.001
**Comorbidities**	0	85 (99%)	1323 (99%)	.863
Hypertension				
Diabetes	0	63 (73.3%)	788 (59.0%)	.009
Anemia	0	55 (64.0%)	828 (62.0%)	.714
Metabolic acidosis	0	22 (25.6%)	240 (18.0%)	.077
Proteinuria	0	11 (12.8%)	227 (17.0%)	.312
Secondary hyperparathyroidism	0	28 (32.6%)	240 (18.0%)	*<*.001
Phosphatemia	0	4 (4.7%)	40 (3.0%)	.39
Heart failure	0	6 (7.0%)	120 (9.0%)	.526
Stroke	0	1 (1.2%)	40 (3.0%)	.506
Conduction and dysrhythmias	0	4 (4.7%)	214 (16.0%)	.005
**Claims-driven features**	0	120 ± 94	109 ± 86	.293
Count of pharmacy claims				
Count of inpatient claims	0	3.85 ± 3.41	3.74 ± 3.62	.773
Count of outpatient claims	0	27.78 ± 24.75	22.07 ± 19.13	.039
Count of professional claims	0	105.37 ± 77.56	87.43 ± 68.02	.039
Net cost of pharmacy claims	0	12 053 ± 11 596	10 440 ± 20 662	.242
Net cost of inpatient claims	0	33 909 ± 53 540	29 440 ± 32 541	.446
Net cost of outpatient claims	0	9354 ± 17 522	8554 ± 17 492	.682
Net cost of professional claims	0	15 512 ± 18 657	11 640 ± 12 748	.061
Range of claims costs	0	11 352 ± 32 606	8852 ± 11 550	.481
Standard deviation of claims costs	0	831 ± 1263	757 ± 806	.593
**Clinical-driven features**	0	17.21 ± 5.46	22.78 ± 5.66	*<*.001
eGFR				
Hemoglobin	3	12.15 ± 2.19	14.25 ± 1.8	*<*.001
Bicarbonate	9	22.9 ± 6.36	25.3 ± 4.22	.001
Serum calcium	6	9.39 ± 3.62	10.21 ± 2.86	.042
Phosphorus	13	3.61 ± 0.87	3.52 ± 0.72	.350
CKD stage 3 duration	0	3.7 ± 0.6	3.9 ± 1.4	.0009
Occurrence of CKD stage 4	0	47 (54.7%)	298 (22.3%)	*<*.001
Occurrence of CKD stage 5	0	42 (48.8%)	277 (20.7%)	*<*.001
Number of emergency department visits	16	2.63 ± 2.18	2.01 ± 1.99	.005

Continuous variables are shown as mean±SD and compared by independent *t*-test (log-transformed if nonnormal); categorical variables are shown as count (percentage) and compared by chi-squared test. “Missing data (%)” indicates the proportion of missing observations for each variable. *P*-values are 2-sided, with *P* < .05 denoting statistical significance. Abbreviations: CKD, chronic kidney disease; ESRD, end-stage renal disease.

Hypertension was prevalent in both groups (99%), but diabetes was more common in ESRD patients (73.3% vs 59.0%, *P* = .009). Secondary hyperparathyroidism and conduction disorders were also significantly higher in the ESRD group. Claims data showed slightly higher outpatient/professional claims counts for ESRD patients (*P* = .039) but no significant cost differences. Clinically, ESRD patients had lower eGFR (17.21 vs 22.78, *P *< .001), lower hemoglobin (12.15 vs 14.25, *P* < .001), and more advanced CKD stages.

### Performance comparison of models across datasets

To facilitate comprehension of model performance across different data sources, we present results using a 24-month observation window, which yielded optimal performance across most models. Tables 2-4 compare the predictive performance of traditional ML and DL models across 3 scenarios: claims data-only, clinical data-only, and merged data.

**Table 2.** Model performance for prediction of ESRD, using claims data-only (24-month observation window, *n* = 1422).

		Model performance metric		
Model type	Model	F1 score	AUROC	AUPRC
Machine learning	Logistic regression	0.33	0.72	0.44
	Random forest	0.36	0.74	**0.48**
	XGBoost	**0.39**	**0.75**	0.47
Deep learning	CNN	0.45	0.82	0.50
	RNN	0.50	0.90	0.52
	LSTM	**0.54**	**0.92**	**0.55**
	GRU	0.50	**0.92**	0.53
	TCN	0.52	0.88	0.53

Bold indicates the best performance within each method category (machine learning or deep learning). Abbreviations: AUPRC, area under the precision-recall curve; AUROC, area under the receiver operating characteristic curve; CKD, chronic kidney disease; CNN, convolutional neural network; ESRD, end-stage renal disease; GRU, gated recurrent unit; LSTM, long short-term memory; RNN, recurrent neural network; TCN, temporal convolutional network.

**Table 3.** Model performance for prediction of ESRD, using clinical data-only (24-month observation window, *n* = 1422).

		Model performance metric		
Model type	Model	F1 score	AUROC	AUPRC
Machine learning	Logistic regression	0.54	0.76	0.47
	Random forest	**0.58**	0.79	0.51
	XGBoost	0.57	**0.80**	**0.52**
Deep learning	CNN	0.56	0.84	0.53
	RNN	**0.61**	0.85	0.53
	LSTM	0.60	**0.88**	**0.56**
	GRU	0.60	0.87	0.55
	TCN	**0.61**	0.83	0.54

Bold indicates the best performance within each method category (machine learning or deep learning). Abbreviations: AUPRC, area under the precision-recall curve; AUROC, area under the receiver operating characteristic curve; CKD, chronic kidney disease; CNN, convolutional neural network; ESRD, end-stage renal disease; GRU, gated recurrent unit; LSTM, long short-term memory; RNN, recurrent neural network; TCN, temporal convolutional network.

**Table 4.** Model performance for prediction of ESRD, using merged data (24-month observation window, *n* = 1422).

		Model performance metric		
Model type	Model	F1 score	AUROC	AUPRC
Machine learning	Logistic regression	0.55	0.75	0.45
	Random forest	0.60	0.84	0.49
	XGBoost	**0.61**	**0.85**	**0.51**
Deep learning	CNN	0.56	0.80	0.46
	RNN	0.62	0.87	0.53
	LSTM	**0.65**	**0.93**	**0.61**
	GRU	0.63	0.90	0.58
	TCN	0.61	0.89	0.58

Bold indicates the best performance within each method category (machine learning or deep learning). Abbreviations: AUPRC, area under the precision-recall curve; AUROC, area under the receiver operating characteristic curve; CKD, chronic kidney disease; CNN, convolutional neural network; ESRD, end-stage renal disease; GRU, gated recurrent unit; LSTM, long short-term memory; RNN, recurrent neural network; TCN, temporal convolutional network.

The results demonstrate several key findings across the different data sources. In the claims data-only scenario, DL models, particularly LSTM (AUROC: 0.92, F1: 0.54) and GRU (AUROC: 0.92, F1: 0.50), substantially outperformed traditional ML approaches such as LR (AUROC: 0.72, F1: 0.33) and RF (AUROC: 0.74, F1: 0.36). For clinical data, while the performance gap narrowed, DL models maintained their advantage, with LSTM achieving the highest performance (AUROC: 0.88, F1: 0.60). Most notably, the merged dataset combining both claims and clinical data yielded the best overall performance, with LSTM achieving better results across all metrics (AUROC: 0.93, F1: 0.65, AUPRC: 0.61). Comprehensive results across observation windows are provided in Appendix S5.

### Key feature analysis using explainable AI techniques

Leveraging the optimal 24-month window and our best ESRD prediction model, we performed (1) cohort-level feature importance to pinpoint the most influential predictors across the population and (2) individual-level SHAP analysis to generate patient-specific explanations of each prediction.

#### Cohort-level key feature identification

**Figure 4.**
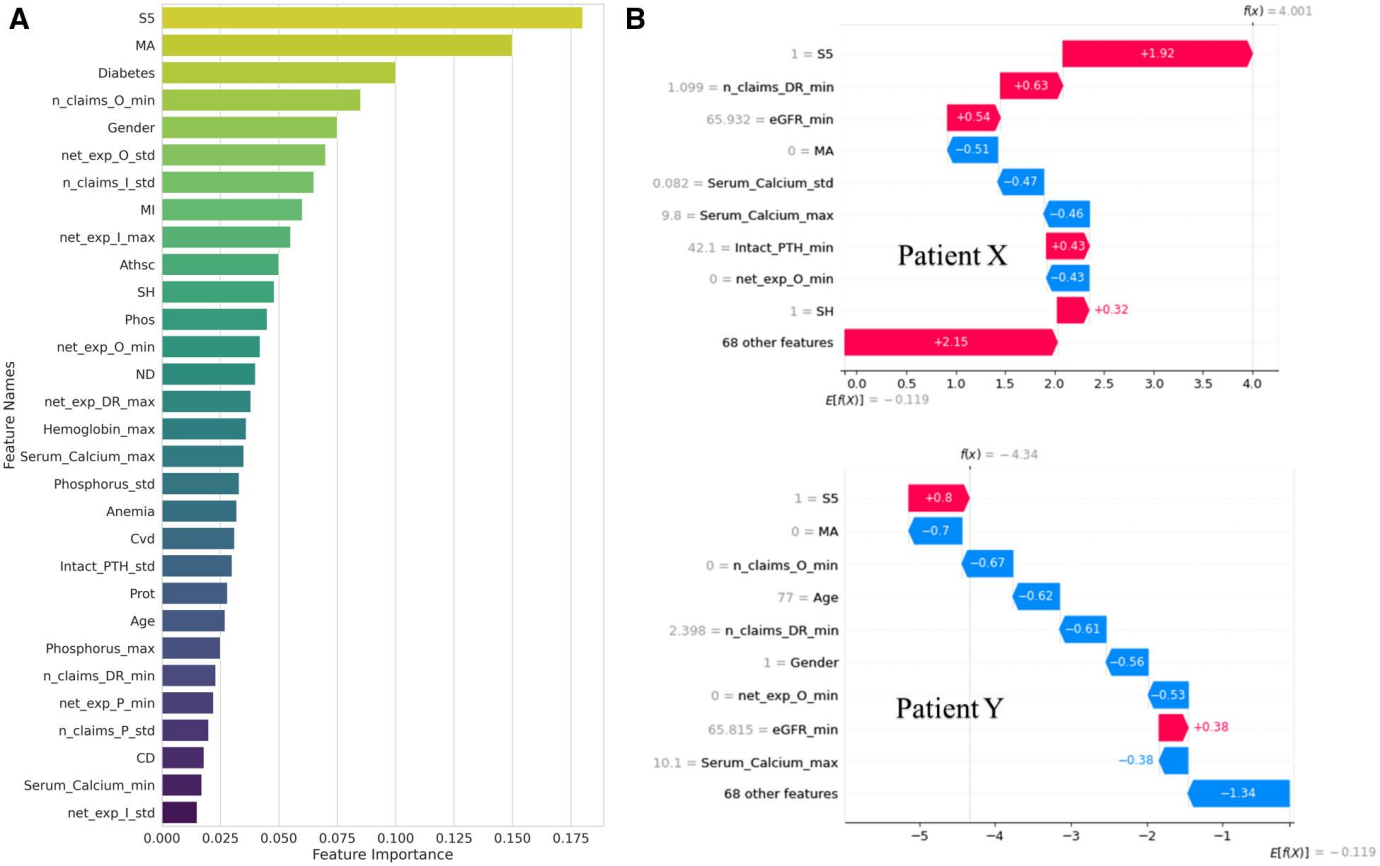
Feature importance and SHAP analysis for ESRD risk prediction using XGBoost model (24-month observation window, *n* = 1422). See Appendix S7 for complete feature names. (A) Top 30 features importance for the XGBoost model. Features are colored from yellow-green (highest importance) to dark blue (lower importance). (B) SHAP analysis demonstrating the variation in feature impact on ESRD risk prediction for 2 patients with CKD stage 5. Red bars indicate features that increase predicted risk, while blue bars indicate features that decrease predicted risk. Abbreviations: CKD, chronic kidney disease; ESRD, end-stage renal disease.

Our feature importance analysis of the optimal ML model, XGBoost, revealed several key predictors for ESRD progression (see Figure 4A). The most critical feature was the presence of CKD stage 5 (S5), aligning with clinical literature and expert feedback on its importance in predicting ESRD. Claims-related features, such as the number of outpatient claims (*n_*claims_*O*) and total inpatient claim expenses (net exp *I*), also ranked highly. The diverse types of top-ranked features underscore the benefit of multisourced data integration, enhancing the model’s predictive power. This combination of clinical and claims data not only improves the accuracy of the predictions but also supports more comprehensive and reliable decision-making in clinical practice. However, while XGBoost’s feature importance reflects the absolute contribution of each feature, it does not specify whether the influence is positive or negative.

#### Feature impact at the individual patient level using SHAP analysis

To support personalized decision-making, we applied SHAP analysis to quantify the features driving each patient’s risk prediction. Figure 4B presents SHAP force plots for 2 correctly predicted high-risk patients, patient X and patient Y, both diagnosed with CKD S5 but exhibiting distinct risk profiles.

For patient X, elevated risk is driven primarily by CKD S5 and a high volume of outpatient claims (*n* claims DR min), while higher eGFR levels and the absence of certain clinical markers mitigate that risk. In contrast, patient Y’s lower risk contribution—despite the same S5 diagnosis—stems from younger age, fewer minimum inpatient claims (net_exp_*O*_min), and lower minimum eGFR values.

### Analysis of model misclassifications: type I and type II errors

**Figure 5.**
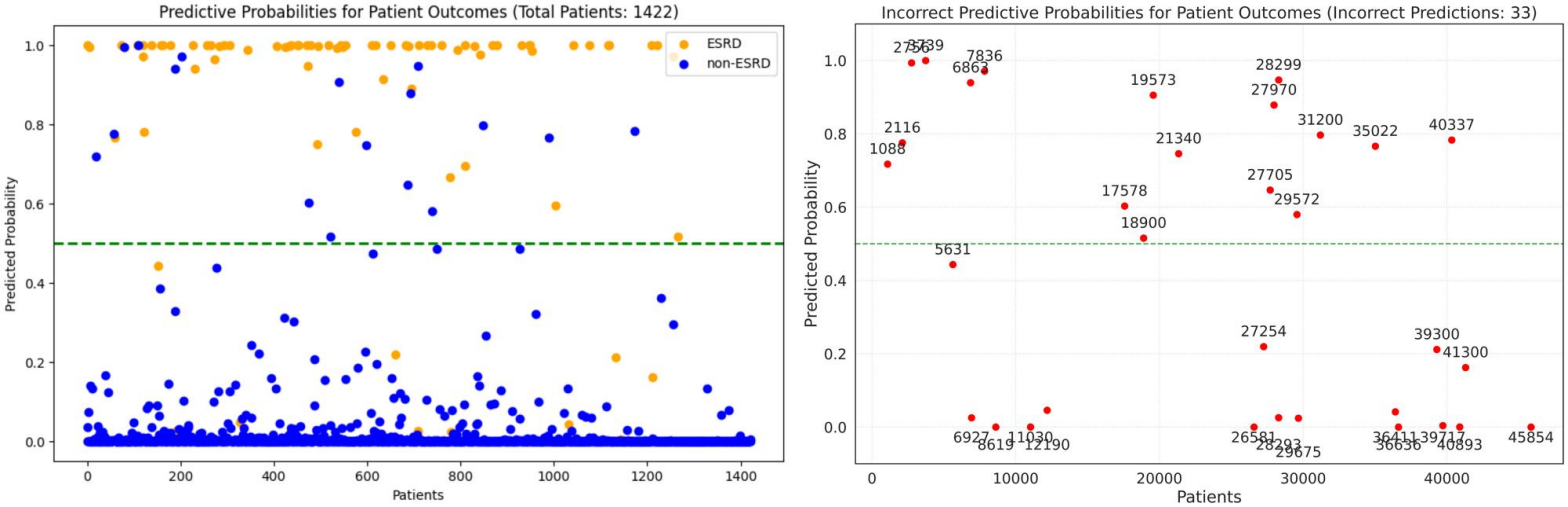
Analysis of model misclassification: type I and type II errors. The left panel shows predicted probabilities for all patient outcomes (24-month conversation window, *n* = 1422), stratified by ESRD (green) and non-ESRD (blue) cases, with patient indices (1-1422) on the *x*-axis. The right panel displays only the incorrectly predicted cases (*n* = 33), using actual patient IDs on the *x*-axis for identification purposes. The horizontal dashed line at 0.5 represents the classification threshold. Abbreviation: ESRD, end-stage renal disease.

All subsequent analyses utilize our optimal 24-month observation window and the LSTM model, which demonstrated superior performance among all tested architectures. To explore these misclassifications, we analyzed type I and type II errors, as depicted in Figure 5. Notably, most incorrect predictions for patients who progressed to ESRD, yet were predicted otherwise, cluster near the lower end of the plot rather than around the 0.5 decision threshold or randomly scattered. This clustering suggests that consistent factors may be influencing these errors.

To understand model misclassification causes, we analyzed type I and type II errors. For type II errors, we compared 16 false negatives (patients incorrectly predicted not to develop ESRD but who did) with 70 true positives (patients correctly predicted to progress). For type I errors, we contrasted 17 false positives (patients incorrectly predicted to develop ESRD) with 1319 true negatives (patients correctly predicted not to progress).

Based on Tables 5 and 6, CKD S5 emerges as a critical feature in prediction errors. For false negatives (incorrectly predicted as not progressing to ESRD), the mean value for CKD S5 at timestamp 6 is significantly lower (0.06 vs 0.36) compared to correctly predicted cases, suggesting these patients did not have an S5 record until later. Conversely, false positives show CKD S5 presence at both timestamps 6 and 7, indicating these patients had S5 records but did not actually progress to ESRD.

**Table 5.** Analysis of type II error (24-month observation window, *n* = 1422): features and their impact on prediction accuracy (feature comparison between false negatives and true positives).

Model	Mean		*P*	Timestamp
	Correct	Incorrect		
*n_*claims_*I*	0.70	0.14	.048752	5
***n*_claims_*O***	5.71	2.67	.001325	2
net_exp_*O*	3831.83	830.61	.002747	0
**S5**	0.36	0.06	.001189	6
**S5**	0.37	0.49	.662073	7
**net_exp_*O***	4219.01	1044.37	.001883	4

This table presents representative subsets of features under various timestamps. Features in bold appear in both analyses.

**Table 6.** Analysis of type I error (24-month observation window, *n* = 1422): features and their impact on prediction accuracy (feature comparison between false positives and true negatives).

Model	Mean		*P*	Timestamp
	Correct	Incorrect		
*n*_claims_DR	13.52	7.21	0.001156	3
***n*_claims_*O***	3.03	1.69	0.009384	4
S4	3831.83	830.61	0.002747	4
**S5**	0.09	0.35	0.000250	6
**S5**	0.08	0.35	0.001388	7
**net_exp_*O***	1044.82	333.61	0.000324	4

This table presents representative subsets of features under various timestamps. Features in bold appear in both analyses.

### Impact of updated eGFR equation on racial bias in predictions

**Figure 6.**
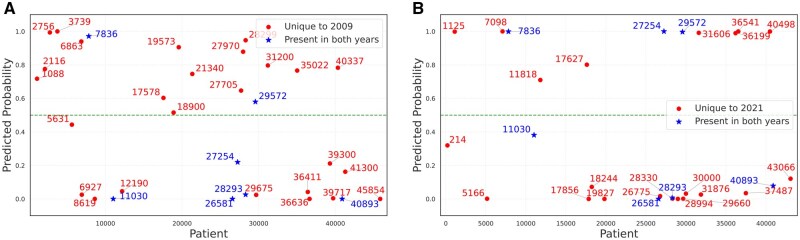
Impact of updated eGFR equations (2009 vs 2021) on prediction errors. Subplots compare incorrect predictions using the 2 eGFR equations, highlighting the differences in misclassification patterns between the traditional and updated formulas. (A) Incorrect predictive probabilities obtained using the 2009 eGFR equation. (B) Incorrect predictive probabilities obtained using the 2021 eGFR equation.

When comparing predictions between the 2009 and 2021 eGFR equations (Figure 6A and B), we observed a decrease in total incorrect predictions from 33 to 28, reducing the overall error rate from 0.0232 to 0.0228. Among these misclassifications, only 7 cases were common to both equations. Analysis of racial distribution in prediction errors revealed varying patterns across different groups: incorrect predictions for White patients increased from 2 to 4, African American patients decreased from 4 to 1, and patients in the “Other” category increased from 10 to 12. However, these numbers are clearly too small for robust insights.

## Discussion

This study demonstrates the value of integrating multiple data sources and DL methodologies for improving ESRD progression predictions in CKD patients. By combining claims and EHR data with LSTM and GRU models, we achieved enhanced predictive accuracy using a 24-month observation window—an optimal balance between early detection and prediction reliability. Our approach addresses a critical gap in chronic disease management by offering a more comprehensive view of patient health trajectories than single-source approaches.

Error analysis revealed important clinical implications. Patients incorrectly predicted not to develop ESRD (false negatives) had significantly lower prevalence of CKD S5 records at timestamp 6 compared to true positives (0.06 vs 0.36), suggesting sudden kidney function decline—a phenomenon clinicians recognize as “crashing” into dialysis. Conversely, false positives showing CKD S5 in consecutive timestamps without progression may reflect data censoring, as patients might have progressed after our dataset endpoint.

While the updated 2021 eGFR equation showed modest improvement in prediction accuracy compared to the 2009 equation (error rate reduction from 0.0232 to 0.0228), the pattern of misclassifications offers greater insight. With only 7 cases misclassified by both equations despite similar overall error rates, these formulas clearly capture different aspects of kidney function. This suggests that while the 2021 equation may better serve minoritized populations by removing race-based adjustments, eGFR alone remains insufficient for accurate ESRD prediction.

These findings underscore the need for a multifaceted approach to risk assessment, combining traditional clinical markers, novel predictive features, and awareness of potential rapid disease progression patterns to develop more targeted interventions across diverse populations.

### Limitations

This study’s reliance on data from a single institution may limit the model’s generalizability to other care settings. Using EHR data introduces observational bias, incomplete records, and underrepresentation of certain patient groups, which can undermine both accuracy and fairness. Although we applied oversampling to mitigate class imbalance, the contrast between high AUROC and lower F1/AUPRC indicates that imbalance remains an issue; more sophisticated approaches—such as ensemble methods, cost-sensitive learning, or hybrid sampling—may be needed. Finally, unaddressed time lags between claims and clinical data can distort temporal relationships and reduce predictive precision.

### Future directions

To address data censoring issues, we will first truncate data to 2016 and analyze outcomes from 2017 to 2018, then expand our dataset beyond 2018 to enhance trajectory modeling. We will integrate unstructured clinical notes to capture patient information missed in structured data, providing a more comprehensive health view and improving prediction accuracy.

We will implement advanced algorithms to synchronize claims and clinical data temporally, reducing time lag effects that currently impact model performance. To understand prediction errors, we will perform SHAP analysis on misclassified cases, identifying key features contributing to these misclassifications and guiding targeted model improvements.

Finally, we will validate our framework’s versatility by applying it to other chronic conditions such as heart disease, assessing its broader potential across various care delivery settings. This systematic expansion will provide actionable insights for both our ESRD prediction model and chronic disease management more broadly.

## Conclusion

This study demonstrates the effectiveness of integrating diverse health-care data with advanced ML techniques to accurately predict ESRD in CKD patients. The combined data approach substantially enhances predictive performance and provides deeper insights into disease progression. SHAP analysis and feature importance assessment highlighted key predictors at both individual and cohort levels.

A critical contribution of this work is a framework for optimizing observation windows, balancing early detection with prediction accuracy. We identified a 24-month observation window as optimal for maximizing predictive effectiveness while minimizing unnecessary interventions. Additionally, evaluating the updated 2021 eGFR equation supports efforts toward health equity and fairer clinical outcomes.

Overall, this research advances CKD management through integrated data and innovative AI methodologies, setting the stage for personalized, equitable care, and future applications in chronic disease prediction and management.

## Supplementary Material

ocaf118_Supplementary_Data

## Data Availability

The data underlying this article consist of active health-care clinical and claims data, which cannot be shared publicly due to privacy regulations and the sensitive nature of the information.
