# Supplementary material for: Enhancing end-stage renal disease outcome prediction: a multisourced data-driven approach
Source: J Am Med Inform Assoc. 2025 Aug 6;33(1):26–36. doi: 10.1093/jamia/ocaf118 (PMC12758457; doi:10.1093/jamia/ocaf118)
Supplement: ocaf118_Supplementary_Data [file ocaf118_supplementary_data.zip › appendix.pdf]

Supplementary

A Detailed Data Source Information

Administrative Claims Data

The de-identified administrative claims dataset used in this study was provided by Highmark, a major health insurance provider serving Pennsylvania, Delaware, and West Virginia. The dataset includes comprehensive records of patient interactions with healthcare professionals from January 1, 2009, to December 31, 2018. The use of this dataset was approved by Highmark's Institutional Review Board.

Clinical EHR Data

The clinical dataset was sourced from the Electronic Health Record (EHR) data of The Nephrology Associates (TMA, P.C.), a leading community nephrology practice serving the greater Pittsburgh region. This dataset contains detailed laboratory results, patient demographics, diagnostic information, and medication records spanning from 1998 to 2018. For consistency of integration with the claims data, we truncated the clinical data to match the 10-year span of the claims data (2009–2018). The usage of the clinical data was approved by the Institutional Review Board of Carnegie Mellon University.

B Laboratory Test Value Ranges for Distribution Detection and Adjustment

Table S1: The table below presents the reference ranges established for laboratory tests during the data preprocessing phase. These ranges were used for distribution detection and adjustment to identify and correct unit inconsistencies.

Laboratory Test	Minimum Value	Maximum Value	Unit Conversion Notes
Serum Creatinine	0	15	$1 \text{mg/dL} = 88.4\mu \text{ mol/L}$
Intact PTH	0	500	$50 \text{pg/mL} = 5.263 \text{pmol/L}$
Hemoglobin	2	20	$1 \text{ g/dL} = 10 \text{ g/L}; 1 \text{ g/dL} = 100/645 \text{ mmol/L}$
Albumin to Creatinine Ratio, Urine	0	600	$1 \text{mg/g} = 1000 \text{mg/mg}$
Bicarbonate (Mostly Arterial)	0	30	$\text{mEq/L} = \text{mmol/L}$
Phosphorus	0	10	$4 \text{mg/dL} = 0.00129 \text{mmol/L}$
Serum Calcium	0	20	$10 \text{mg/dL} = 0.00250 \text{mmol/L}$
Urine Albumin	0	600	-
eGFR	0	150	-

C Comorbidity Feature ICD Code Prefixes

Table S2: Comorbidity features with ICD-9 and ICD-10 code prefixes used for feature extraction in this study

Condition	ICD-9 Prefixes	ICD-10 Prefixes
Diabetes	249*, 250*	E08*–E13*
Hypertension	401*–405*	I10*–I15*
Cardiovascular Disease	429*	I25*
Anemia	285.9	D64.9, D50*–D59*
Metabolic Acidosis	276.2	E87.2
Proteinuria	791.0	R80.9
Secondary Hyperparathyroidism	252.02, 588.81	E21.1, N25.81
Phosphatemia	275.3	E83.39
Atherosclerosis	440*	I70*
Congestive Heart Failure (CHF)	428*	I50*
Conduction & Dysrhythmias	426*–427*	I44*–I49*, Z450*, Z958*
Myocardial Infarction (MI)	410*	I21*
CVD Other	390*–459*	I05*–I09*, I30*–I52*
Stroke	433*–434*	I63*
Fluid/Electrolytes	276*	E86*–E87*
Mineral Disorders	275*	E20*–E21*, E83*, N25*
Nutritional Deficiencies	260*–269*	E40*–E46*, E50*–E64*, D50*
CKD Stage 4	585.4*	N18.4*
CKD Stage 5	585.5*	N18.5*
CKD End-Stage Renal Disease (ESRD)	585.6*	N18.6*

Note: prefix * matches any sequence of trailing digits. For example, I44*–I49* captures all codes from I440 through I499 such as I4402 and I4439; Z450* captures Z4502, Z45010, Z45018, etc.

D Neural Network Implementation Details

Hardware Specifications

Resource	Specification
GPU	2 × NVIDIA RTX 4090 (24GB VRAM)
RAM	512GB DDR4
Storage	2TB NVMe SSD

Table S3: Hardware specifications used for model training and evaluation.

Software Environment and Library Versions

Component	Version	Purpose
Python	3.10.8	Programming language
CUDA	11.6	GPU acceleration library
PyTorch	1.13.0	Deep learning framework
H2O	3.38.0.1	Machine learning model tuning
NNI	2.10	Neural architecture search
scikit-learn	1.1.3	Traditional ML algorithms
pandas	1.5.1	Data manipulation and analysis
NumPy	1.23.4	Numerical computing

Table S4: Software environment and library versions used for model implementation.

Model Architecture, Hyperparameters, and Training Details

Model	Architecture Details	Parameters	Hyperparameters	Training Time
CNN	3 conv layers (64, 128, 256 filters), 2 FC layers (512, 256)	4.3M	Batch size: 32, Optimizer: Adam, Learning rate: 1e-4, Dropout: 0.3	8.5 hours
RNN	2 recurrent layers (128 units), 2 FC layers (256, 128)	2.1M	Batch size: 64, Optimizer: RMSprop Learning rate: 5e-4, Dropout: 0.2	9.2 hours
LSTM	2 LSTM layers (256 units), 1 FC layer (128)	5.7M	Batch size: 32, Optimizer: Adam Learning rate: 1e-4, Dropout: 0.3	15.3 hours
GRU	2 GRU layers (256 units), 1 FC layer (128)	4.9M	Batch size: 32, Optimizer: Adam Learning rate: 1e-4, Dropout: 0.3	13.7 hours
TCN	4 temporal blocks, kernel size 3, 128 filters	3.8M	Batch size: 32, Optimizer: Adam Learning rate: 2e-4, Dropout: 0.2	12.1 hours

Table S5: Model architecture, hyperparameters, and training details for each neural network model.

E Model Performance Metrics Across Observation Windows

Table S6: Comparison of AUROC and F1 scores across different models, using claims data-only, clinical data-only, and merged data (24-month observation window, n=1,422). Bold font is used to mark the best performance within each category (ML or DL methods), while bold and italic font highlights the best overall performance across all methods (ML + DL).

(a) Performance of models using claims data only.

			**6 months**		**12 months**		**18 months**		**24 months**		**30 months**	
		Model/Metrics	AUC	F1	AUC	F1	AUC	F1	AUC	F1	AUC	F1
Claims Data Only Modeling	Machine Learning Methods	Logistic Regression	0.61	0.24	0.64	0.31	0.70	0.32	0.72	0.33	0.71	0.29
		Random Forest	0.69	0.26	0.73	0.33	0.74	0.41	0.74	0.36	0.68	0.33
		XGBoost	0.70	0.27	0.75	0.35	**0.78**	**0.42**	0.75	0.39	0.69	0.38
	Deep Learning Methods	CNN	0.72	0.36	0.73	0.42	0.81	0.44	0.82	0.45	0.84	0.44
		RNN	0.77	0.39	0.75	0.44	0.83	0.49	0.90	0.50	0.82	0.47
		LSTM	0.76	0.33	0.79	0.43	0.86	0.45	***0.92***	***0.54***	0.87	0.44
		GRU	0.70	0.34	0.78	0.46	0.88	0.45	0.91	0.50	0.86	0.42
		TCN	0.72	0.36	0.81	0.37	0.85	0.46	0.88	0.52	0.85	0.43

(b) Performance of models using clinical data only.

			**6 months**		**12 months**		**18 months**		**24 months**		**30 months**	
		Model/Metrics	AUC	F1	AUC	F1	AUC	F1	AUC	F1	AUC	F1
Clinical Data Only Modeling	Machine Learning Methods	Logistic Regression	0.63	0.46	0.70	0.49	0.76	0.51	0.76	0.54	0.75	0.53
		Random Forest	0.70	0.53	0.76	0.53	**0.80**	0.58	0.79	0.58	0.77	0.54
		XGBoost	0.73	0.52	0.76	0.55	**0.80**	**0.59**	**0.80**	0.57	0.72	0.55
	Deep Learning Methods	CNN	0.73	0.51	0.75	0.54	0.80	0.55	0.84	0.56	0.80	0.52
		RNN	0.74	0.53	0.77	0.57	0.82	0.60	0.85	0.61	0.79	0.55
		LSTM	0.77	0.54	0.78	0.56	***0.84***	0.63	***0.88***	0.60	0.82	0.57
		GRU	0.80	0.54	0.77	0.57	0.83	0.61	0.87	0.60	0.84	0.53
		TCN	0.72	0.50	0.73	0.53	0.80	0.57	0.83	0.61	0.81	0.55

(c) Performance of models using merged data.

			**6 months**		**12 months**		**18 months**		**24 months**		**30 months**	
		Model/Metrics	AUC	F1	AUC	F1	AUC	F1	AUC	F1	AUC	F1
Merged Data Modeling	Machine Learning Methods	Logistic Regression	0.65	0.46	0.69	0.47	0.76	0.54	0.75	0.55	0.78	0.54
		Random Forest	0.74	0.44	0.77	0.52	0.83	0.58	0.84	0.60	0.80	0.55
		XGBoost	0.73	0.45	0.76	0.50	0.82	0.58	**0.85**	**0.61**	0.82	0.59
	Deep Learning Methods	CNN	0.70	0.45	0.76	0.46	0.77	0.55	0.80	0.56	0.82	0.55
		RNN	0.75	0.51	0.80	0.58	0.86	0.60	0.87	0.62	0.85	0.61
		LSTM	0.76	0.53	0.84	0.59	***0.93***	0.63	***0.93***	***0.65***	0.84	0.59
		GRU	0.75	0.51	0.76	0.56	0.91	0.62	0.90	0.63	0.86	0.57
		TCN	0.71	0.49	0.74	0.57	0.88	0.60	0.89	0.61	0.91	0.59

F Distribution of time to ESRD for the ESRD cohort

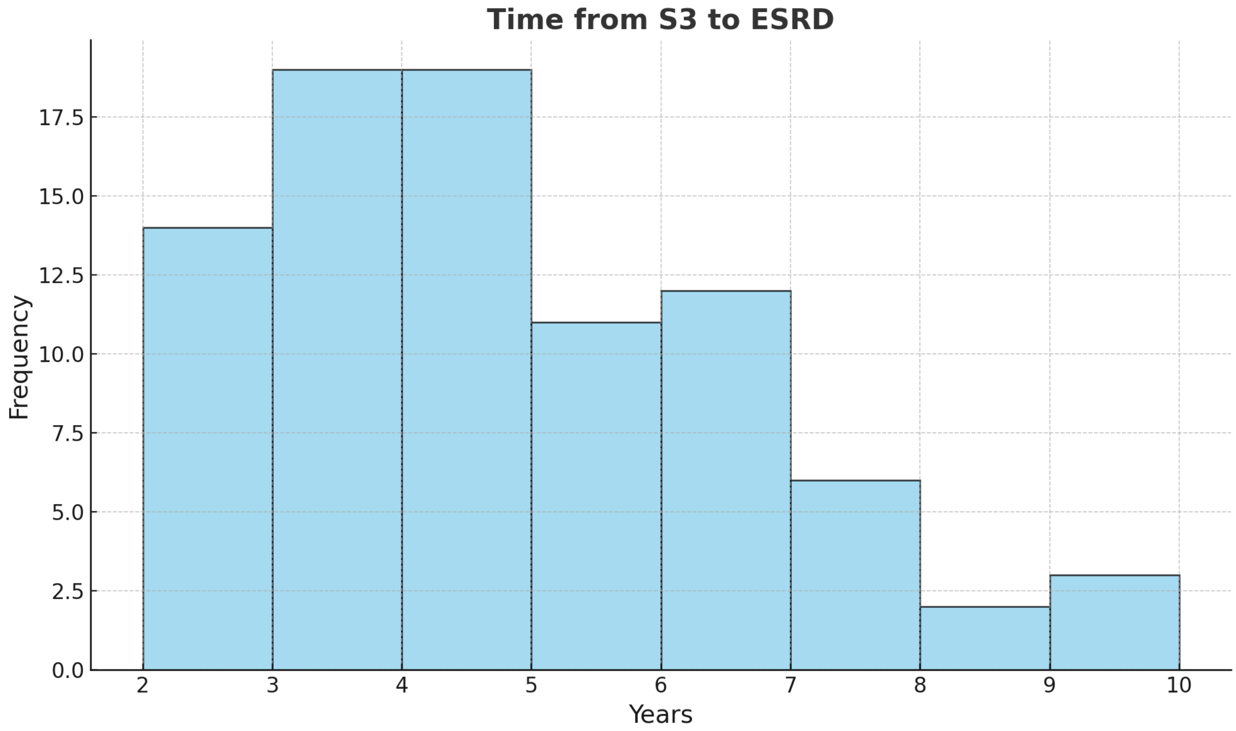

Figure S1: The plot illustrates the variability in time to ESRD across the cohort, with a mean time of 4.82 years and a standard deviation of 1.82 years.

G Feature Abbreviations

Table S7: Mapping between feature abbreviations in The feature importance plot and corresponding full names.

**Short Name**	**Full Name**
S5	CKD Stage 5
MA	Metabolic Acidosis
Diabetes	Diabetes
n_claims_O_min	Number of Outpatient Claims (Min)
Gender	Gender
net_exp_O_std	Net Expense for Outpatient Claims (Std Dev)
n_claims_I_std	Number of Inpatient Claims (Std Dev)
MI	Myocardial Infarction
net_exp_I_max	Net Expense for Inpatient Claims (Max)
Athsc	Atherosclerosis
SH	Secondary Hyperparathyroidism
Phos	Phosphatemia
net_exp_O_min	Net Expense for Outpatient Claims (Min)
ND	Nutritional Disorders
net_exp_DR_max	Net Expense for Drug-Related Claims (Max)
Hemoglobin_max	Hemoglobin Level (Max)
Serum_Calcium_max	Serum Calcium Level (Max)
Phosphorus_std	Phosphorus Level (Std Dev)
Anemia	Anemia Diagnosis
Cvd	Cardiovascular Disease
Intact_PTH_std	Intact Parathyroid Hormone (Std Dev)
Prot	Proteinuria
Age	Age
Phosphorus_max	Phosphorus Level (Max)
n_claims_DR_min	Number of Drug-Related Claims (Min)
net_exp_P_min	Net Expense for Pharmacy Claims (Min)
n_claims_P_std	Number of Pharmacy Claims (Std Dev)
CD	Conduction & Dysrhythmias
Serum_Calcium_min	Serum Calcium Level (Min)
net_exp_I_std	Net Expense for Inpatient Claims (Std Dev)
